# An Enclosed Nest Can Increase the Risk of Predation on Adults

**DOI:** 10.1002/ece3.74168

**Published:** 2026-08-07

**Authors:** Yu Zhang, Huiyue Jiang, Yunwei Li, Kevin H. Mayo, Duo Liu, Mingju E

**Affiliations:** ^1^ School of Life Sciences Changchun Normal University Changchun China; ^2^ Department of Biochemistry, Molecular Biology and Biophysics University of Minnesota Health Sciences Center Minneapolis Minnesota USA

**Keywords:** cavity‐nesting birds, nest predation, parental investment, snakes

## Abstract

Even though enclosed nests provide a physical barrier for cavity‐nesting birds, nest predation is the primary cause of breeding failure and a major driver of avian population dynamics. Previous studies have largely focused on the loss of eggs and nestlings, whereas the predation risk faced by adult birds during the nestling‐rearing stage has received far less attention. We recently documented a predation event in which an Amur rat snake ( *Elaphe schrenckii* ) remained concealed in a nest box after consuming nestlings of the Daurian Redstart ( *Phoenicurus auroreus* ) and captured a returning male. This observation provides empirical evidence for understanding predation risks in enclosed nesting environments and suggests that adult mortality may warrant consideration in predation risk assessment. Additional observations are needed to determine whether similar predation events occur in other cavity‐nesting systems.

## Graphical abstract

We document a rare predation event where an Amur rat snake, after consuming nestlings inside an enclosed nest box of the Daurian Redstart, remained concealed to ambush and capture a returning male parent. This finding reveals that enclosed nests can become lethal traps for adult birds, highlighting an overlooked cost of cavity‐nesting strategies.

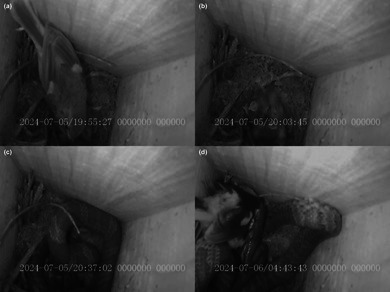

## 1 Introduction

Reproductive success is fundamental to the maintenance of bird populations and is largely determined by the nesting environment that effectively ensures offspring survival (Heenan 2013; Mainwaring et al. 2014). Bird breeding in open‐cup nests benefits from a wider field of environmental surveillance for parents; however, the exposure of such nests increases the risk to both nestlings and adults from climatic stress and predation (Marques 2013). In contrast, enclosed nests (e.g., cavity and domed nests) provide a safer microenvironment that buffers nestlings against abiotic stressors, such as from wind and rain (Mainwaring et al. 2023; Martin et al. 2017), as well as providing some protection from predators (Martin and Li 1992).

Nevertheless, nest predation remains the principal cause of reproductive failure in birds that use enclosed nests (Ibáñez‐Álamo et al. 2015; Lima 2009). Snakes are among the primary nest predators for cavity‐nesting birds (DeGregorio et al. 2014; Liu et al. 2025). Due to their highly sensitive olfactory and infrared sensory ability, snakes can locate concealed nests and prey on them (Shine and Sun 2003). Once detected, altricial nestlings face extremely high mortality rates due to their limited mobility. In addition, the nest's fixed location and restricted field of view substantially reduce the ability of adult birds to detect and evade potential predators (Collias 1997), that increases the risk of entrapment and mortality during nest predation.

Female birds typically invest more in reproduction than males. In particular, incubation and nocturnal brooding result in females spending substantially more time at the nest, thereby potentially exposing them to a higher risk of predation (Adamík and Král 2008; Diniz 2011). Nevertheless, during the nestling stage, both male and female parents face an increased risk of predation due to frequent feeding trips to the nest (Martin et al. 2000; Skutch 1949). Although many studies to date have focused on nest predation of incubating adults and/or nest contents (Ibáñez‐Álamo et al. 2015), the survival risks faced by adult birds breeding in enclosed nests during the nestling‐rearing stage have received less attention.

**FIGURE 1:**
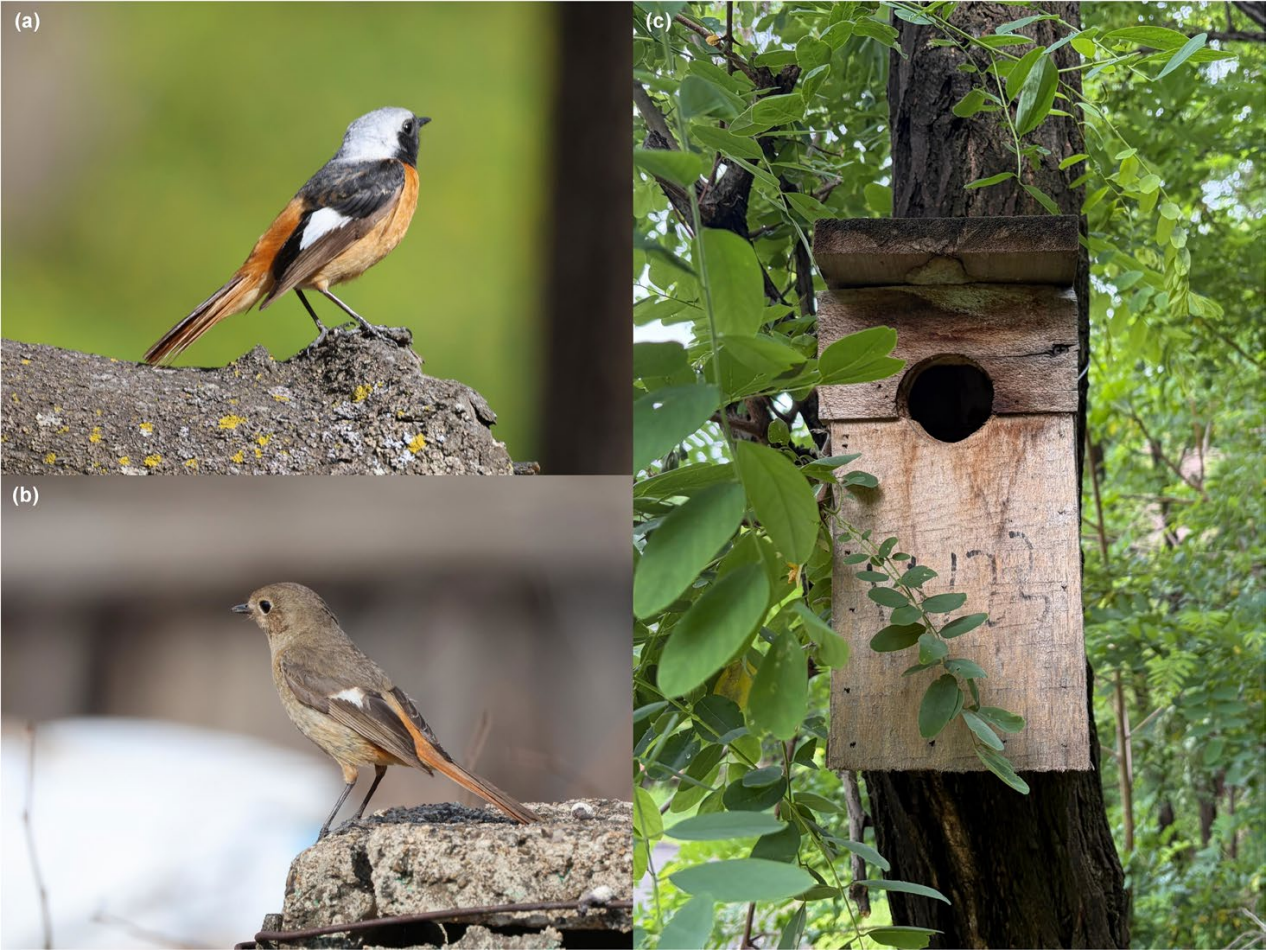
Daurian Redstarts and the nest box used in this study. (a) Adult male; (b) adult female; (c) external view of the artificial nest box. Photos by Zhengqi Shi.

The Daurian Redstart *Phoenicurus auroreus* (Figure 1a,b) is a cavity‐nesting species and widely distributed across eastern Eurasia (Collar 2020). During the breeding season, parental roles are divided such that the female is solely responsible for incubation, whereas both parents participate in providing food and rearing the nestlings (Wan et al. 2023). On July 5, 2024, we recorded a predation event using video monitoring: a male Daurian Redstart was captured by a snake inside its nest box, after the snake had preyed on the nestlings.

## 2 Methods

This study was conducted in the Qingchui Peak National Forest Park, Chengde City, Hebei Province, China (40°54′–42°40′ N, 117°11′–119°15′ E). We randomly selected tree species, tree height, and nest entrance orientation for the installation of artificial nest boxes. The nest boxes were positioned 2–3 m above ground, and the entrance hole size was 4.5 cm in a circular shape. Nest boxes have these dimensions: 26 cm (height) by 11 cm × 11 cm. Entrances were located on the upper one‐third of the front wall of the nest box (Figure 1c). A total of 112 artificial nest boxes were installed in the study area. Beginning in mid‐April, artificial nest boxes within the study plots were monitored weekly to record breeding bird species and associated reproductive parameters. Once Daurian Redstarts were confirmed to have entered the nestling‐rearing stage, their parental provisioning behavior was monitored using a portable video camera (DSJ‐F7; Yingweida Technology Co. Ltd.).

## 3 Results

**FIGURE 2:**
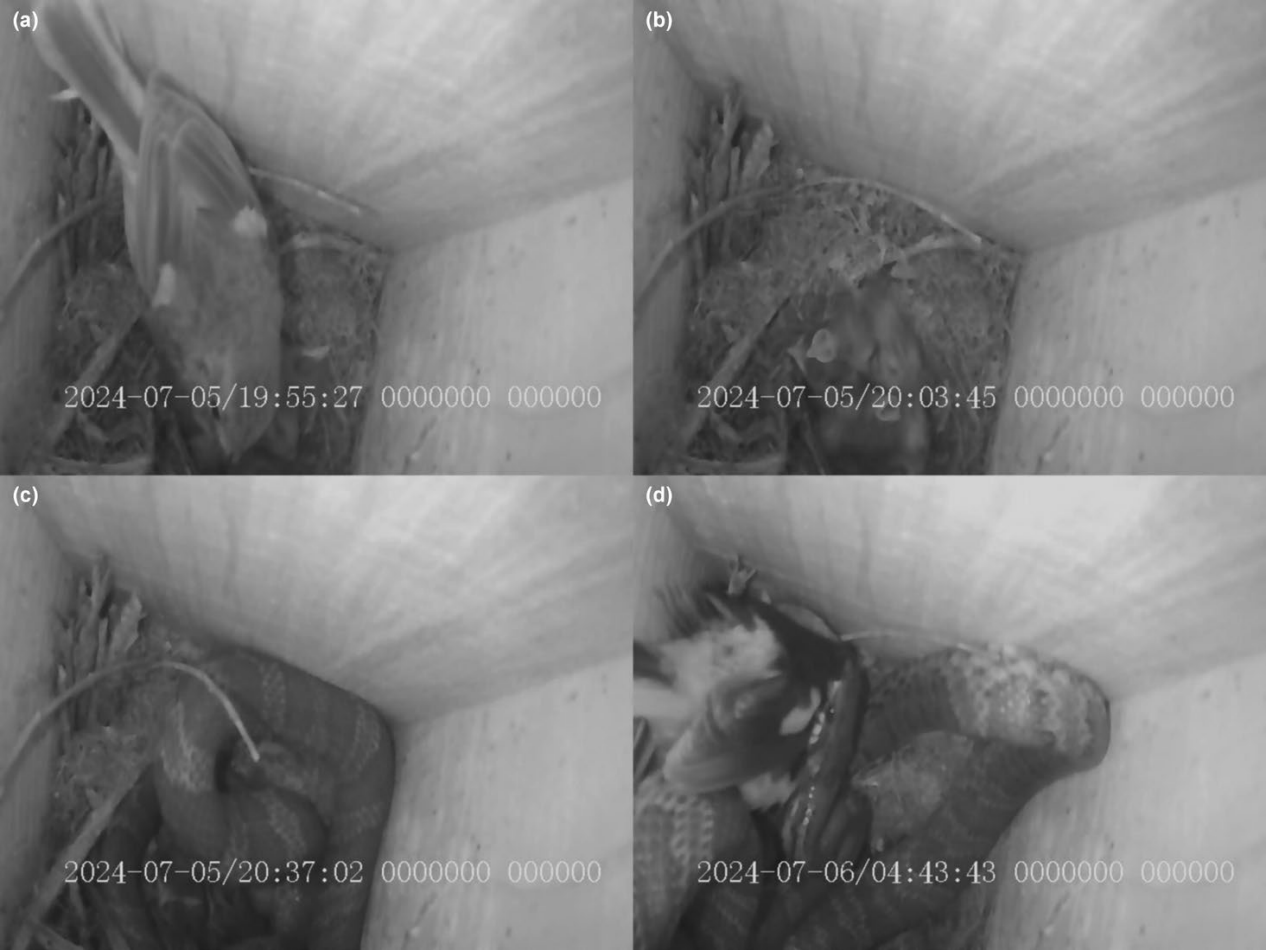
Images associated with predation by an Amur rat snake on a Daurian Redstart nest. (a) The female Daurian Redstart made her last recorded provisioning visit to the nest box (details in Video S1); (b) nestlings exhibited normal begging behavior prior to snake entry (details in Video S2); (c) the snake preyed on Daurian redstart nestlings in the nest box (details in Video S3); (d) the snake dragged the male Daurian redstart into the nest box and swallowed it (details in Video S4).

On July 5, 2024, while monitoring parental feeding of two‐day‐old Daurian Redstart nestlings in nest box W23, we recorded a predation event by a snake. The nest box was monitored continuously from 16:57:55 on 4 July 2024 to 08:51:44 on 6 July 2024. Video footage showed that the female Daurian Redstart last entered the nest box at 19:55:13 to provision the nestlings (Figure 2a and Video S1). Following this visit, the nestlings continued to exhibit normal begging behavior (Figure 2b and Video S2). At 20:36:35, an Amur rat snake ( *Elaphe schrenckii* ) entered the nest box. At the time the snake entered the nest box, the female was not inside the nest box. Between 20:37 and 20:43, the snake consumed all nestlings in the box (Figure 2c and Video S3). After predation, the snake coiled and rested inside the nest box, remaining there until early morning of the next day. At 03:30:28, the snake briefly left the nest but returned ~19 min later (03:49:58) and again coiled inside the nest. At 04:42:05, the snake suddenly lunged toward the nest box entrance, at which point the audio recording captured distinct sounds of flapping wings. Subsequently, the snake rapidly dragged an adult bird into the box and consumed it (Figure 2d and Video S4). Image analysis identified the prey as a male Daurian Redstart returning to the nest to deliver food. Males were identified by their conspicuous white wing patches and strong contrast between pale crowns and dark backs, as females display fainter color contrast and smaller wing patches. By the end of the video monitoring period at 08:51:43, the snake remained inside the nest box in a stationary state.

## 4 Discussion

Our observation of this event highlights an important adaptive trade‐off associated with enclosed nest structures. While the physical concealment of such nests can effectively reduce the risk of nestling predation, their single, fixed entrance can also function as a potential trap (Christman 1997; Nilsson 1984). In contrast to open‐nesting species, where adults benefit from a wide field of view that facilitates early threat detection and escape, cavity‐nesting birds face compromised visibility and constrained escape options when inside the nest (Collias 1997). Furthermore, when they are outside the nest, the adult birds may be unable to detect ambush predators concealed within the enclosed nest. This represents a clear vulnerability, as starkly illustrated in the present predation event.

In this instance, the snake did not leave immediately after consuming the nestlings, but rather occupied the nest for an extended period of time that substantially increased the likelihood that the returning parents would be ambushed (DeGregorio et al. 2015). The female was absent from the nest box when the snake entered. A possible explanation is that she sensed the threat and chose not to return to the nest. In contrast, the male returned to provision the nestlings and was depredated, presumably because he did not detect the predator. The snake remained in the nest box for hours, which transformed a single nest predation event from a transient opportunity into a sustained trap, potentially increasing its overall impact on reproductive success.

This event documents the continuous sequence from nestling predation to the subsequent predation of an adult bird, providing empirical evidence for understanding predation risk within enclosed nesting environments. For parent birds, continued care for offspring involves a trade‐off between reproductive investment and exposure to predation risk (Montgomerie and Weatherhead 1988). In this context, even adaptive nest structures may sometimes impose unexpected costs on breeding adults, potentially creating ecological traps under particular circumstances. This study suggests that the pressure of nest predation is not limited to reproductive output (i.e., the loss of offspring), but may also directly result in the mortality of adult breeding birds. Notably, as this incident was observed in an artificial nest box, more observational records are needed to determine whether similar predation events occur in natural cavities.

## Author Contributions

**Yu Zhang:** conceptualization (equal), data curation (equal), writing – original draft (equal). **Huiyue Jiang:** formal analysis (equal), investigation (equal), methodology (equal). **Yunwei Li:** data curation (equal), formal analysis (equal), writing – original draft (equal). **Kevin H. Mayo:** visualization (equal), writing – review and editing (equal). **Duo Liu:** methodology (equal), supervision (equal), writing – review and editing (equal). **Mingju E:** conceptualization (equal), funding acquisition (equal), writing – review and editing (equal).

## Funding

This work was supported by the Scientific Research Fund of Jilin Provincial Education Department JJKH20261560 to M.E. and the National Natural Science Foundation of China (grant no. 32272650 to Duo Liu).

## Ethics Statement

All birds were captured under a bird‐ring license issued by the China Bird Banding Center. This field survey was approved by Qingchui Peak National Forest Park. All experimental procedures were reviewed and approved by the Animal Research Ethics Committee of Changchun Normal University (approval number: 2025006). There are no records of deaths or nest destruction as a result of this study.

## Conflicts of Interest

The authors declare no conflicts of interest.

## Supporting information


**Video S1:** The last recorded provisioning visit of a female Daurian Redstart to the nest box before the predation event.

**Video S2:** Normal begging behavior of Daurian Redstart nestlings before the entry of an Amur rat snake into the nest box.

**Video S3:** Predation of Daurian Redstart nestlings by an Amur rat snake inside the nest box.

**Video S4:** The Amur rat snake dragging the male Daurian Redstart into the nest box and consuming the adult bird.

## Data Availability

All data supporting the findings of this study are available within the article and its Supporting Materials. Video recordings of the complete predation event (nestling predation and adult ambush) are provided as Videos S1–, S4.
